# A Straightforward, Sensitive, and Reliable Strategy for Ethyl Carbamate Detection in By-Products from Baijiu Production by Enzyme-Linked Immunosorbent Assay

**DOI:** 10.3390/foods13121835

**Published:** 2024-06-11

**Authors:** Zifei Wang, Qing Liu, Jiaqi Luo, Pengjie Luo, Yongning Wu

**Affiliations:** 1 Research Unit of Food Safety, NHC Key Laboratory of Food Safety Risk Assessment, Ministry of Health, China National Center for Food Safety Risk Assessment, Chinese Academy of Medical Sciences (2019RU014), Beijing 100021, China; wangzifei@cfsa.net.cn (Z.W.); liuqing@cfsa.net.cn (Q.L.); wuyongning@cfsa.net.cn (Y.W.); 2 State Key Laboratory of Environmental Chemistry and Ecotoxicology, Research Center for Eco-Environmental Sciences, Chinese Academy of Sciences, College of Resources and Environment, University of Chinese Academy of Sciences, Beijing 100085, China; jqluo_st@rcees.ac.cn

**Keywords:** ethyl carbamate, enzyme-linked immunosorbent assay, Baijiu by-products, fermentation and distillation

## Abstract

Baijiu is a renowned Chinese distilled liquor, notable for its distinctive flavor profile and intricate production process, which prominently involves fermentation and distillation. Ethyl carbamate (EC), a probable human carcinogen, can be potentially formed during these procedures, thus prompting significant health concerns. Consequently, the contamination of EC during Baijiu production has become an increasingly pressing issue. In this study, we developed a rapid and easily operable immunoassay for determining EC in the fermented materials used in Baijiu production. The development of a high-quality antibody specific to EC facilitated a streamlined analytical procedure and heightened method sensitivity. Furthermore, we systematically evaluated other essential parameters. Following optimization, the method achieved an IC_50_ value of 11.83 μg/kg, with negligible cross-reactivity against EC analogs. The recovery study demonstrated the method’s good accuracy and precision, with mean recovery rates ranging from 86.0% to 105.5% and coefficients of variation all below 10%. To validate the feasibility of the technique, we collected and analyzed 39 samples simultaneously using both the proposed immunoassay and confirmatory gas chromatography–mass spectrometry (GC-MS). A robust correlation was observed between the results obtained from the two methods (R^2^ > 0.99). The detected EC levels ranged from 2.36 μg/kg to 7.08 μg/kg, indicating an increase during the fermentation process.

## 1. Introduction

Ethyl carbamate (EC), a genotoxic and multi-site carcinogenic substance, can be formed during the production of fermented foods and alcohol beverages [1,2]. While it has been previously employed in the treatment of leukemia and other ailments, numerous studies have demonstrated its carcinogenic risk to humans [3,4]. Building upon prior research findings, the international Agency for Research on Cancer upgraded EC classification from Group 2B to Group 2A (probably carcinogenic to humans) [5]. Although the presence of EC in fermented foods and alcohol beverages has been frequently documented [2,5], higher concentrations are primarily associated with alcoholic liquor, particularly distilled spirits [4,5]. Consequently, researchers have highlighted the risks associated with consuming EC-contaminated alcohol beverages, especially among heavy drinkers [6,7,8]. The Joint Food and Agriculture Organization (FAO)/WHO Expert Committee on Food Additives had also concluded that alcohol beverages were the major contributors to EC exposure for human beings [9]. The highest EC levels are typically found in spirits derived from stone fruits [4,10]. To enhance monitoring of EC contamination in alcohol beverages, various governmental authorities have established allowable limits for EC in some alcohol beverages. The Canadian government initially established an acceptable EC value in distilled liquor at 150 μg/L. Subsequently, other countries such as the USA, Japan, and the Czech Republic also instituted EC limits for distilled spirits and other alcohol beverages [6].

Baijiu, one of the most famous distilled liquors worldwide, has dominated the Chinese alcoholic beverage market for decades [11]. This traditional distilled spirit is noted for its complex making process, including fermentation and distillation. During the aforementioned processes, EC can be generated via two pathways: the chemical reactions involving ethanol and cyanate, and the microbial reactions occurring during fermentation (such as arginine’s metabolism to urea). Other factors such as thermal processing and metal transition, which are routinely involved in Baijiu production, can also contribute to EC formation [5,12,13]. The contamination of ethyl carbamate (EC) in Baijiu has been a subject of extensive reporting. Significant variability in EC levels has been noted, which correlates with the distinct production regions and categories of Baijiu [13]. Notably, Strong-Flavor Baijiu tends to exhibit higher EC concentrations. In comparison to other global distilled spirits, a considerable number of Baijiu samples exceed the Canadian regulatory limit of 150 μg/L for EC in distilled spirits [12]. Additionally, risk assessment studies emphasize the imperative for enhanced contamination control measures specifically targeting Strong-Flavor Baijiu [6,13]. Consequently, it is necessary to closely monitor EC contamination in the food supply of the Baijiu industry. Up to now, no allowable limit of EC has been set for Baijiu and its by-products.

For EC determination in alcoholic beverages, gas chromatography–mass spectrometry (GC-MS) and high-performance liquid chromatography (HPLC) are generally used [12,14,15,16]. Their usage give confidence in the aspects of good sensitivity and repeatability. However, the sophisticated instruments and the high cost of the analysis possibly limit their applications in rapid, on-site, and high-throughput fields. Recently, the immunoassay, based on the principle that an antigen can bind to its specific antibody, has been gradually introduced for EC analysis in different types of alcoholic beverages, such as Chinese rice wine [17] and red wine [18,19]. Nevertheless, to achieve fast and reliable detection of EC in the matrices containing various interferents, researchers still face some ongoing challenges. First, the lack of distinctive chemical groups of EC probably hamper the successful production of an antibody with high quality and affinity against EC, which can decrease the sensitivity of the method [17]. Second, the presence of interfering compounds in the complex sample matrix may give rise to an overestimated EC level and unreliable results [16,20]. To address these issues, various approaches have been taken for more robust and sensitive EC quantification. For example, under an acidic environment, 9-xanthydrol is usually used to react with EC to form xanthyl ethyl carbamate, a derivative containing aromatic rings. This modified hapten can help increase the efficiency of antibody production [17]. However, the stability of the synthesized derivative, the lengthy sample preparation time, and the repeatability of the derivatization reaction should be taken into account [21]. Some new nanoparticles have also been designed to obtain more precise and reliable EC measurement in some liquid matrices, such as red wine [18], flavored liquor [22], Huangjiu [23], and Baijiu [24]. Unfortunately, these strategies may not be suitable for the in situ analysis of EC in solid and complex fermented matrices from the Baijiu industry. So far, to the best of our knowledge, there is no immunoassay—particularly the ELISA technique—for EC quantification in fermented by-products from Baijiu production.

The present project aimed to develop a rapid, easily operated, and sensitive enzyme-linked immunosorbent assay for EC detection in Jiupei and Jiuzao from the Baijiu industry. Initially, a high-affinity antibody against EC was designed and produced, significantly enhancing the simplicity and repeatability of the analysis. To extract EC from the tested matrices with less organic solvent and fewer procedures, different extraction systems were evaluated, followed by optimization of the essential parameters for ELISA performance. The avoidance of the derivatization process ensured the convenience of the whole analytical operation. Under the optimized conditions, the proposed method demonstrated favorable sensitivity, specificity, and accuracy. To evaluate the application of the method, a total of 39 real samples were collected and analyzed by the immunoassay and GC-MS. The results suggested a good correlation between the two methods. Overall, this method fosters scientific innovation in the detection and controlling of EC in by-products from the Baijiu industry.

## 2. Materials and Methods

### 2.1. Reagents and Apparatus

The standard solutions of EC and EC-d_5_ (deuterium ethyl carbamate, acted as the internal standard for GC-MS analysis) were acquired from A Chemtek (Worcester, MA, USA); the purity was >99%. The goat anti-mouse immunoglobulin (IgG, 31.0 mg/mL), bovine serum albumin (BSA), ovalbumin (OVA), Tween-20, and Proclin300 were supplied by Sigma-Aldrich (St. Louis, MO, USA). The anti-EC monoclonal antibody (mAb, 5.0 mg/mL) and coating antigen (4.0 mg/mL) were prepared in our laboratory. Other chemical reagents, such as dimethyl formamide (DMF), 1-ethyl-3-(3-dimethylaminopropyl)-carbodiimide (EDC), N-hydroxysuccinimide (NHS), and ethyl carbonochloridate, were of analytical grade and purchased from Aladdin Chemical Technology Co., Ltd. (Shanghai, China).

The ELISA microplate reader was purchased from Thermo Scientific (Hudson, NH, USA). The absorbance value was measured at a wavelength of 450 nm. The microtiter plates (flat bottom, 96-well, 0.3 mL/well) were from WDWK Biotech (Beijing, China). The GC-MS determination was conducted by gas chromatography (GC, 7890A) combined with the 5977A mass spectrometer (MS, Agilent Technologies, Santa Clara, CA, USA). The solid phase extraction (SPE, PRiME^®^ HLB cartridge) column was also from Agilent.

### 2.2. Collection of Samples

A total of 39 real samples, including the samples involved in the fermentation process (Jiupei), and the foodstuffs obtained after the distillation step (Jiuzao), were all purchased from Baijiu industry suppliers. The Light-Flavor and Strong-Flavor Baijiu types were chosen in this study. The Light-Flavor Jiupei samples were collected every two days up to the 14th day. The samples of Strong-Flavor Jiupei were collected at intervals of 10 days, extending up to a period of 50 days. These samples are the by-products such as distiller’s grains and fermentation mash during Baijiu production. All the samples were treated as follows: first, they were dried in an oven at 65 °C overnight, followed by being ground and mixed thoroughly. Then, the mixtures were sealed and kept at 4 °C for further analysis. The moisture of the samples was calibrated according to the guidelines and principles stated in the corresponding section of ISO 6469 [25].

### 2.3. Preparation of Hapten

**Figure 1:**
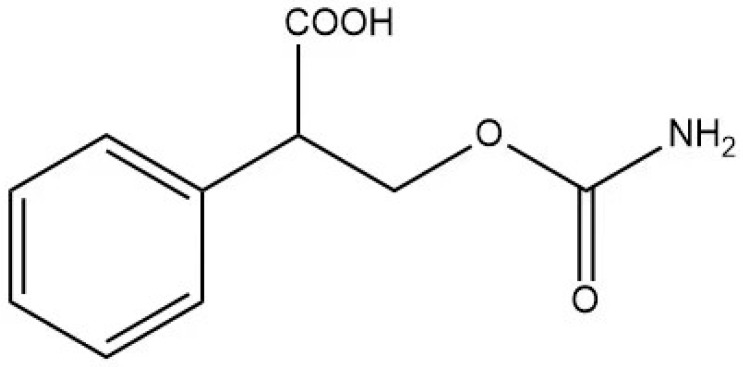
The chemical structure of the modified EC hapten (3-carbamoyloxy-2-phenylpropanoic acid).

This research utilized a hapten, 3-carbamoyloxy-2-phenylpropanoic acid, which was synthesized in our laboratory. The chemical structure of this compound is depicted in Figure 1. Furthermore, we utilized HPLC, LC-MS, and ^1^H NMR techniques for the structural characterization of the synthesized hapten. The experimental apparatus, conditions, and detailed explanations can be found in Appendix A appended at the end of the article.

### 2.4. Preparation of Antigen

First, the BSA solution was prepared by dissolving 100 mg of BSA into 16 mL of the pre-prepared buffer solution in our lab (1.35 g of Na_2_HPO_4_·12H_2_O, 0.3 g of NaH_2_PO_4_·2H_2_O, 8 g of NaCl, all dissolved in deionized water and subsequently adjusting the volume to 500 mL with the same deionized water, then adjusted the pH value to about 7.4.), and stirring at 37 °C for 8~10 h. Then, the newly produced BSA protein solution was added into the activated hapten solution for coupling the hapten with the carrier protein. This hapten solution was prepared by dissolving 2.8 mg of hapten, 270 mg of NHS, and 460 mg of EDC in 2 mL of DMF under the conditions of 4 °C in advance. To improve the efficiency of conjugation, 4 mL of NaOH solution (2.5 M) was added into the above-mentioned solution, and the pH was adjusted to 7.0~7.2. After reaction for 30 min, dialysis was performed by transferring the reacted solution to 0.01 mol/L PB (phosphate solution, comprising 0.46 g of KH_2_PO_4_ and 2.38 g of Na_2_HPO_4_·12H_2_O dissolved in deionized water; the volume is then adjusted to 1 L with additional deionized water, with the pH level being modified to 9.6) at 4℃. The dialyzed solution was stirred for about 24 h, during which the dialyzed solution was changed every 6 h. When the UV scanning results showed no small molecule absorption peak, it was centrifuged to take the supernatant, namely the successfully conjugated EC antigen. This prepared compound was kept in a centrifuge tube and stored at −20 °C. The UV absorption spectra of the EC hapten, carrier protein, and artificial antigen were scanned, and the change of the maximum absorption peak in the spectrum identified successful conjugation between the carrier protein and the EC hapten (3-carbamoyloxy-2-phenyl-propanoic acid). In this study, the monoclonal antibodies were generated through the immunization of Balb/c male mice with a synthesized artificial antigen, following standard laboratory protocols.

### 2.5. Sample Preparation

First, 1 g of the sample was mixed with 2 mL of acetonitrile and 0.4 g of sodium chloride. Then, the mixture was dispersed by vortex mixing for 1 min, then subjected to sonication for 10 min. Subsequently, the mixture was centrifugated at 8000× *g* for 10 min, and the supernatant was evaporated to near dryness with nitrogen at 60 °C. The residue was re-dissolved in 500 μL of the optimal reconstitution solution. After about 1 min of vortex mixing, 50 μL of the solution was used for further ELISA analysis.

For GC-MS detection, the sample preparation was based on our previous research and slightly modified [10]. To begin with the test, 40 μL of the d_5_-EC was spiked onto the sample, then a designated amount of distilled water (3 mL) and sodium chloride (1 g) was added and mixed. After that, 4 mL of acetonitrile was added to the mixture, followed by ultrasonic extraction for 10 min and centrifugation at 9000× *g* for 15 min. Later, 2 mL of the upper layer was transferred to the selected SPE column. Then, the column was washed twice with 1 mL of ultra-pure water. The EC was eluted twice with 1 mL of acetonitrile/methanol mixture [4:1 (*v*/*v*)]. The supernatant was totally collected and filtered with a 0.22-µm syringe filter, and then submitted to GC-MS for further analysis.

### 2.6. ELISA Testing Procedures

In this study, the determination of EC was performed based on the principle of indirect competitive ELISA [26]. First, 100 μL of the diluted coating antigen was pipetted into the individual wells of the microtiter plate. The plate was covered with a lid and incubated for 2 h at 37 °C. Then, the coating solution was aspirated from all the wells and discarded. After washing with 250 μL of buffer solution three times, 150 μL of blocking buffer was added to block the remaining protein-binding sites. The incubation condition was set at 37 °C for 1 h. To start the competitive reaction, the diluted standard solution (0.01 mol/L PB for the negative wells), the sample solution, and the specific primary antibody were added to the target well. The reaction lasted 25~30 min at 37 °C. After removing the liquor in the wells, the plate was washed as in the above-mentioned step. The secondary antibody was prepared at an optimal concentration, and 100 μL of this antibody was added to each well for incubation at 37 °C for 1 h. Afterwards, 100 μL of the substrate solution was added, and the enzyme-based reactions lasted at least 20 min in the dark. The reaction was stopped by adding 50 μL of 1 M HCl to each well. Lastly, the plate was read on the microplate reader set to 450 nm for HRP-based substrate development.

### 2.7. Optimization of Pretreatment

#### 2.7.1. Extraction for EC

In accordance with the results of previous research [9,14,24,27], acetonitrile, *n*-hexane, ethyl acetate, and methylene chloride were selected in this study to explore their extraction efficiency for EC. Specifically, 1 mL of standard solution (EC, 500 μg/L) was mixed with 2 mL of the extraction solvent for the positive wells (when acetonitrile was used, sodium chloride would be added in the extraction system) and 0.01 mol/L PB was used for the negative wells. Following dissolution by vortex mixing for 1 min and sonication for 15 min, 1 mL of the organic phase was aspirated and dried under a gentle stream of nitrogen gas at 60 °C. The residue was reconstituted to 1 mL with 0.01 mol/L PB solution. The rest of the analytical steps were similar to those mentioned in the sections of “Extraction“ and “ELISA testing procedures”. The absorbance value of the wells free of EC (A_max_) and the inhibitory effect/ratio were used to interpret the results. For evaluation of the inhibitory effect/ratio, an inhibitory curve was established by setting the logarithmic values of the EC standard concentrations as the values of *x*-axis, and the B/B_0_ as the values of *y*-axis. In this study, B/B_0_ typically represents the ratio of absorbance in the samples with the concentrations of EC as same as those without EC. The inhibition effect was calculated as follows: Inhibition rate (%) = (1 − B/B_0_) × 100.

#### 2.7.2. Reconstituted Solution

This study utilized refined pretreatment protocols and a competitive ELISA detection method to evaluate the efficacy of diverse reconstituted analyte solutions. For evaluation of the reconstituted solution, 0.01 mol/L PB, 10% acetone, and 1% glycerin were analyzed, and the A_max_ and inhibitory effect were used to assess the results.

### 2.8. Optimization for ELISA Analysis

#### 2.8.1. Checkboard Analysis

The checkboard titration was conducted to explore the most appropriate concentrations for the antibody and the corresponding coating antigen. The coating antigen solutions were diluted to 1:5000, 1:20,000, 1:400,000, and 1:100,000, as well as for the primary antibody solutions (1:5000, 1:10,000, 1:50,000, and 1:100,000). Then, the EC standard was diluted to the appropriate concentration and acted as the competitor for the icELISA analysis, and 0.01 mol/L PB solution was added in the wells as the negative control. The A_max_ and the inhibition rate were used to assess the results.

#### 2.8.2. The Optical Concentration of the Secondary Antibody, the Sample, and the Primary Antibody

The A_max_ and IC_50_ value were used for the evaluation. IC_50_ is often defined as the concentration of the competitive substance that leads to 50% of the B/B_0_ based on the inhibition curve [24]. For the secondary antibody, different dilutions (1:1000, 1:2000, 1:5000) were set to achieve the best results. The most suitable ratio of the sample to the primary antibody was also assessed: 20 μL of the sample and 80 μL of the primary antibody, 40 μL of the sample and 60 μL of the primary antibody, 50 μL of the sample and 50 μL of the primary antibody, and 70 μL of the sample and 30 μL of the primary antibody were tested under the optimized conditions.

### 2.9. The Impact of Alcohol Strength

For EC detection, the alcohol concentrations can affect the robustness and sensitivity of the analytical method [2]. A previous study indicated a good linear relationship in an alcohol strength range of between 5% and 65% in the tested samples [28]. The results from another HPLC analysis revealed that an alcohol strength ranging from 38% to 42% can improve the derivatization efficiency [14]. Similar conclusions were reached by other studies; the acceptable alcohol concentrations were from 20% to 40% [23,29,30]. For a study performed by ELISA, a series of alcohol concentrations were tested: 2%, 5%, 10%, and 20%. The results indicated that the IC_50_ steadily rose when the alcohol strength increased correspondingly. Furthermore, an improper alcohol concentration may influence the stability of the EC derivative [17]. In this study, the samples, fortified with two different levels (10 μg/kg and 50 μg/kg) were analyzed under the different alcohol strengths of 2%, 10%, 20%, and 40%, and the recovery rate was used to evaluate the results.

### 2.10. Performance Parameters for the Method

#### 2.10.1. Sensitivity

For the sensitivity evaluation, the blank samples were spiked with a series of EC standard concentrations at 0, 1, 3, 9, 27, and 81 μg/kg, and subsequently analyzed by the optimized test steps to construct the standard curves (*n* = 6). The standard curve was established by recording the results from the ELISA reader. The logarithmic concentrations of EC standard solution and the B/B_0_ values were plotted as the *x*-axis and the *y*-axis, respectively. The limit of detection (LOD) was defined as the mean of 20 blank samples plus threefold of the standard deviation, and the limit of quantitation (LOQ) was measured as the mean plus tenfold of the standard deviation.

#### 2.10.2. Specificity

To investigate the specificity of the newly developed method, the cross-reactivity (CR) values of three EC analogs, namely methyl carbamate, butyl carbamate, and acrylamide, were measured; the CR was defined as the IC_50_ ratio of EC to its analogs according to the established standard curve.

#### 2.10.3. Accuracy and Precision

The typical blank samples were employed for the accuracy and precision evaluation. For accuracy, the recovery rates were tested by spiking the EC-free samples at three levels (5, 10, and 50 μg/kg). The precision evaluation was performed based on the coefficient of variation (CV) calculation of the intra- and inter-day studies. Intra-day repeatability was estimated by the results of six successive extractions, and inter-day reproducibility was presented by the results of the same samples over six continuous days.

### 2.11. Confirmatory Analysis

For the confirmatory analysis, all the samples were analyzed using an Agilent 7890A gas chromatograph equipped with an Agilent 5977 mass spectrometer under the selected ion monitoring mode. The capillary VF-WAX column (30 m length × 0.25 mm i.d., 0.39 µm film thickness; J&W, Folsom, CA, USA) was used in this study. Hydrogen was applied as a carrier gas at a constant flow rate of 1.0 mL/min. The temperature program was set as follows: 60 °C held for 1 min; linear heating to 180 °C at 8 °C/min; 240 at 10 °C/min; and 240 °C held for 5 min. The injector and detector temperatures were set at 250 °C and 270 °C, respectively. On the selected ion mode condition, m/z values of 62, 74, and 89, and 64, 76, and 94 were defined as the main ions for EC and d_5_-EC identification, respectively. The *m*/*z* values of 62 and 64 were chosen as the quantitative ions for EC and d_5_-EC, respectively.

## 3. Results and Discussion

### 3.1. Identification of the Hapten and Antigen

**Figure 2:**
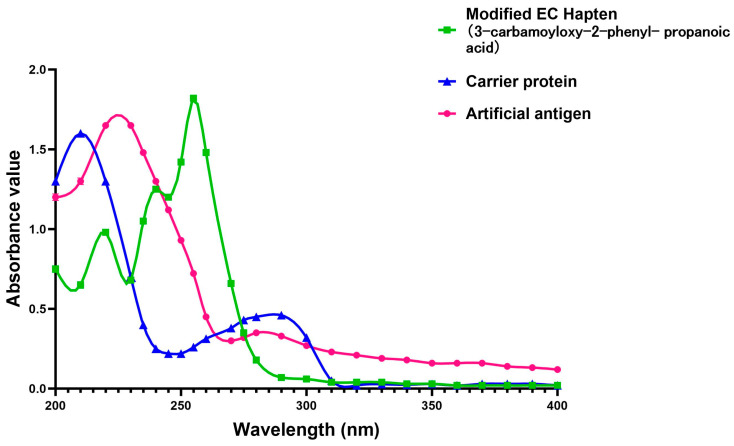
The ultraviolet-visible spectrum of the modified EC hapten, bovine serum albumen (BSA) as the carrier protein, and the purified artificial antigen towards EC (conjugated by BSA and the modified EC hapten).

**Figure A1:**
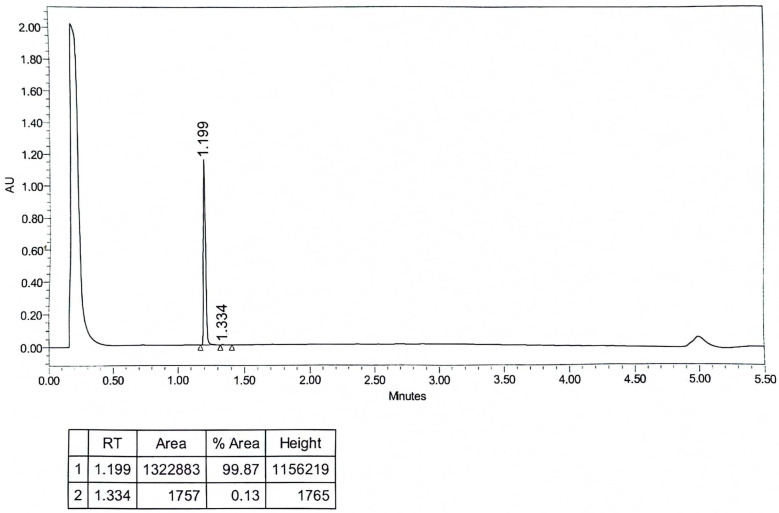
The HPLC spectrogram of the hapten towards EC.

**Figure A2:**
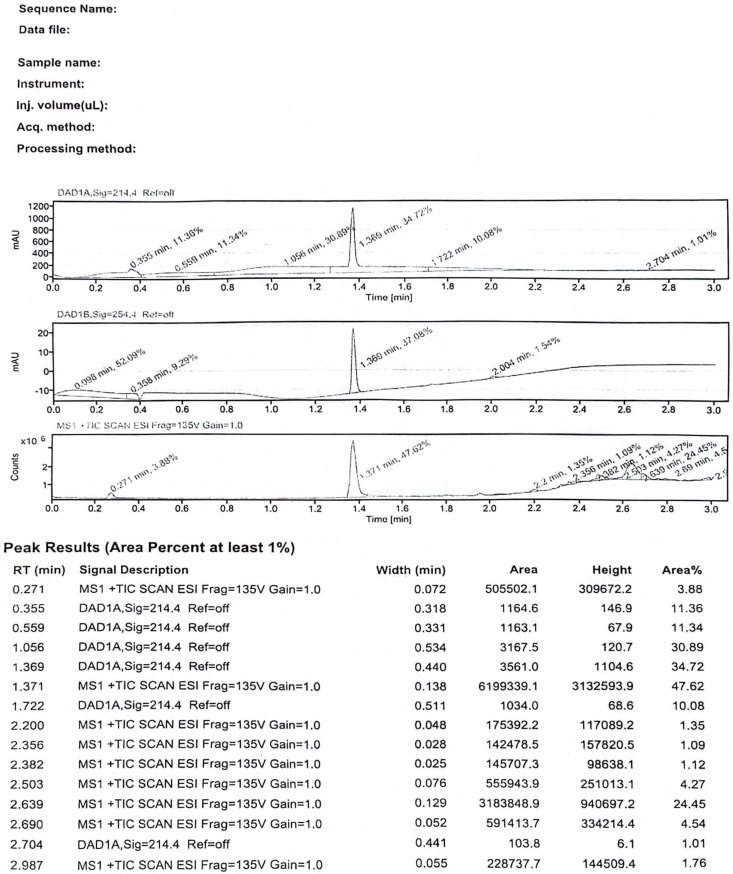
Identification of 3-carbamoyloxy-2-phenyl-propanoic acid by LC−MS.

**Figure A3:**
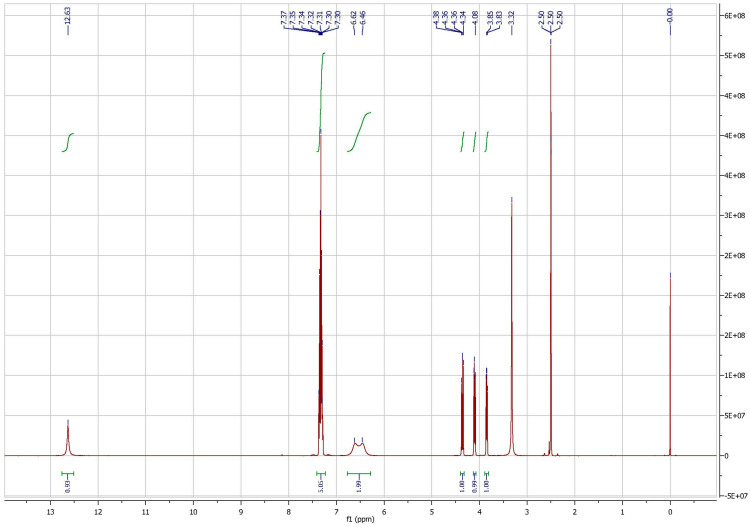
Identification of 3-carbamoyloxy-2-phenyl-propanoic acid by ^1^H NMR.

Because of the lack of characteristic groups and the low molecular weight (89.09 g/mol), researchers have taken different approaches to improve the affinity and the sensitivity of the produced antibody against EC [9,22,23]. The reagent 9-xanthydrol was often involved in the pre-analysis step to introduce the aromatic chemical structure to the artificial hapten and correspondingly increase the molecular weight of designed hapten [17,18]. Nevertheless, this reaction could complicate the whole analytical procedure and raise the amount of organic solvents. Furthermore, the stability of the EC derivative should also be considered, which may have a negative effect on the repeatability and reliability when real samples are tested. Taken together, the process of derivatization was avoided in this study, and the modification of EC with a compound containing an aromatic ring was introduced. The chemical structure of the synthesized hapten, specifically 3-carbamoyloxy-2-phenylpropanoic acid, is illustrated in Figure 1 and was identified by HPLC, LC-MS, and ^1^H NMR. According to the results in Appendix A (Figure A1, Figure A2 and Figure A3), the hapten towards EC was successfully synthesized for the further steps. After the conjugation between the hapten and the carrier protein, the characteristic absorption peaks of haptens, carrier proteins, and artificial antigens were obviously shifted (Figure 2), indicating the successful combination between the EC hapten and the carrier protein, BSA.

### 3.2. Optimization of Organic Extraction of EC from Fermented Sample

**Figure 3:**
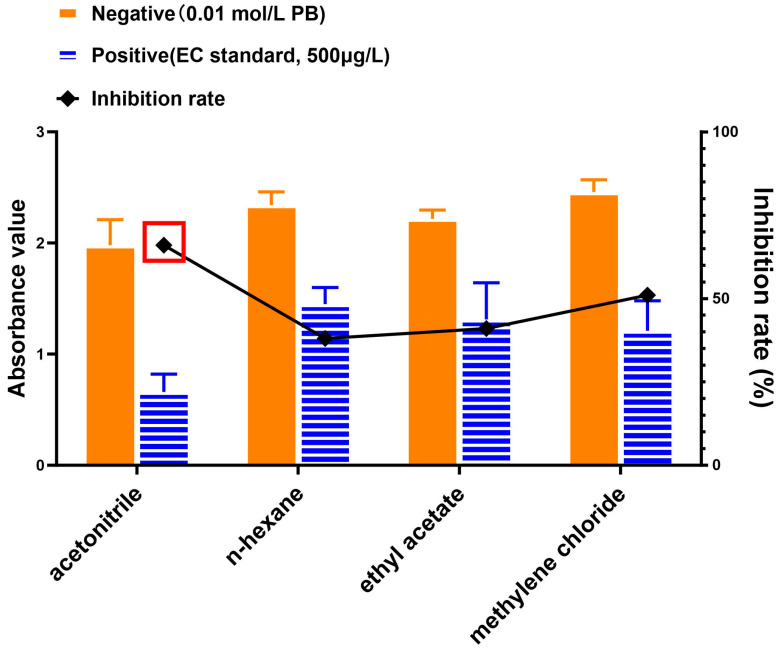
Comparison of EC extraction as followed by competitive immunoassay. Acetonitrile, n-hexane, ethyl acetate, and methylene chloride were employed to extract EC from fermented mash that contained added EC standard (labeled “Positive”) or phosphate buffer (PB; labeled “Negative”). The competitive immunoassay inhibition rate for each sample was calculated as the % difference of the negative and positive absorbency values. The red box indicates the best result for the given experimental conditions.

Fermented material or “mash” from the Baijiu industry is a rich source of protein, minerals, and fiber [31,32]. Yet mash matrix is the most likely source of EC despite it being a valuable byproduct that is typically repurposed as an animal feed [32]. The complexity of this matrix may pose a detrimental impact on either the robustness and/or accuracy in testing for EC [33]. With regards to this complexity and the added need for detection of potentially trace levels of EC, an extraction method with fewer steps and minimal usage of organic solvent was explored. Given the polarity and chemical structure of EC, acetonitrile, methylene chloride, n-hexane, and ethyl acetate were selected for comparison of extraction efficiency [9,14,17,19,34] for the EC standard added (“Positive”) to fermented mash (phosphate buffer or PB was added to fermented mash to serve as a negative control or background which was labeled “Negative”). Extraction efficiency was followed using our newly developed competitive immunoassay where lower absorbencies (or higher inhibition rate) indicated a higher presence of EC. As shown in Figure 3, although the absorbance for acetonitrile background was on the low side (indicating a relatively slight false-positive amount for competitive inhibition of the immunoassay), the acetonitrile extracted sample yielded the most inhibited signal:noise ratio as well, thus indicating the highest inhibition rate relative to the other solvents. Therefore, acetonitrile was chosen as the extraction solvent for EC in the tested matrices from Baijiu.

### 3.3. Comparison of Solutions for Reconstitution of Extracted EC

**Figure 4:**
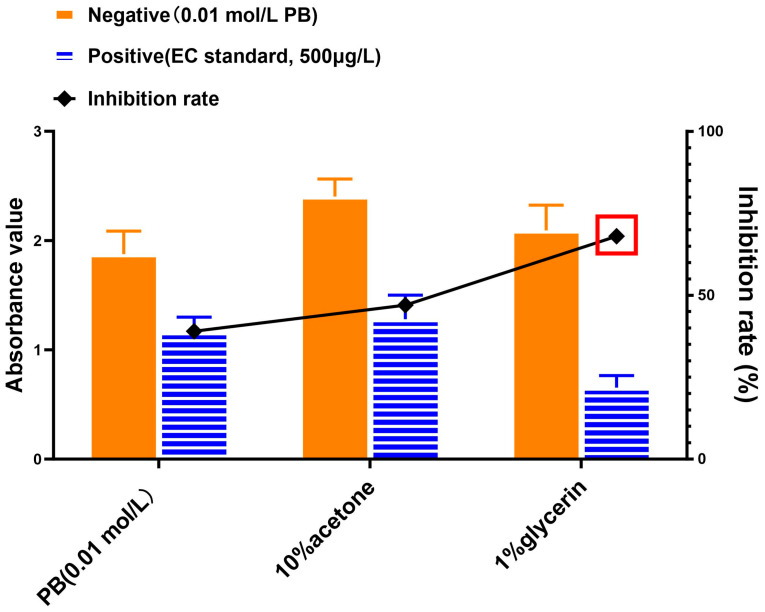
Comparison of reconstitution solutions for extracted EC as followed by competitive immunoassay. PB (phosphate buffer), 10% acetone, or 1% glycerin were used to resuspend EC-extracted samples and recovered EC was followed by competitive immunoassay. The competitive immunoassay inhibition rate for each sample was calculated as the % difference of the negative and positive absorbency values. The red box indicates the best result for the given experimental conditions.

Different solutions were tested for maximizing the response of matrix-extracted EC. As with the previous section, our developed competitive immunoassay was employed to compare results, but in this case for the comparison of resuspension of EC-extracted samples. As displayed in Figure 4, the absorbencies for all tested resuspension agents were similar for “Negative” (background samples) whereas the absorbency for the 1% glycerol was the lowest (best inhibition) for the “Positive” sample. In addition, the inhibition rate of 1% glycerol solution was the highest among all the reconstituted solutions. As a result, 1% glycerol was selected to reconstitute extracted EC samples.

### 3.4. Effect of Alcohol Concentration

Present studies found that a 20~40% alcohol strength in analyzed matrices did not affect the analytical performance for EC determination [14,28]. However, for the immunoassay, a previous study indicated that an alcohol concentration of 2% was preferable for EC detection in Chinese rice wine [17]. In our research, under alcohol concentrations of 2~40%, the recovery rates were acceptable and the inhibitory effects were not significantly different (*p* > 0.05, Table 1). In conclusion, the influence of alcohol concentration on the proposed ELISA technique can be neglected.

### 3.5. Optimization for ELISA Conditions

#### 3.5.1. Checkboard Titration Test

Prior studies have suggested that a value of A_max_ from 1.7 to 2.2 combined with the higher inhibitory effect in the checkboard experiment, often led to better sensitivity of the methods [35,36]. Moreover, the consumption of primary antibody should be as little as possible under similar analytical conditions due to the methods’ cost-effectiveness [17]. Therefore, a dilution of 1:40,000 for the coating antigen and a dilution of 1:50,000 for the primary antibody were applied in the determination process (Table 2).

#### 3.5.2. Consumption of the Secondary IgG Antibody, the Sample and the Primary Antibody

In the analysis, a series of dilutions of 1:1000, 1:2000, and 1:5000 for the secondary IgG antibody were tested. In Table 3, when the IgG antibody was diluted to 1:1000, the lower IC_50_ and desired A_max_ value were obtained, indicating the preferred dilution of IgG for the EC determination.

The ratio of the sample to the primary antibody was further investigated based on the similar parameters such as A_max_ and IC_50_. The results were acceptable when 40 μL of the sample and 60 μL of the primary antibody, or 50 μL of the sample and 50 μL of the primary antibody was used (Table 4). However, in view of the lengthy time and the high expenditure involved for antibody production and identification, the ratio of the sample to the primary antibody was finally set at 50:50.

### 3.6. Analytical Performance for ELISA

#### 3.6.1. Sensitivity

**Figure 5:**
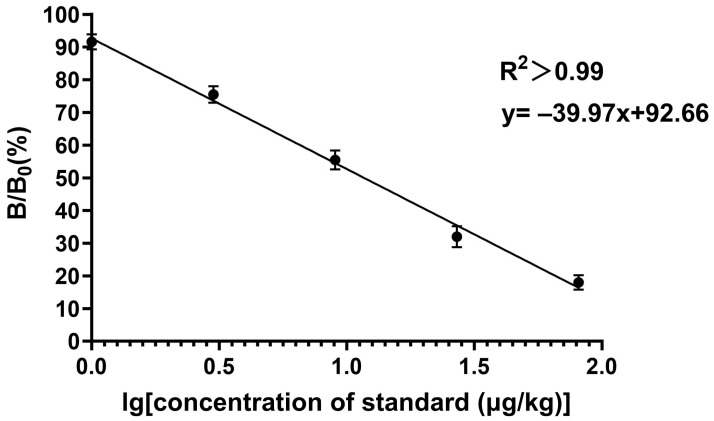
The standard curve of EC in fermented materials from Baijiu. In this experiment. B and B_0_ are utilized to indicate the absorbance measurements for the positive and negative wells, respectively.

The fundamental principle of the developed method is the competitive reaction between EC and the coating antigen towards the limited sites on the primary antibody. The calibration curve was constructed by spiking a series of the EC standard onto the blank samples, which had been confirmed by the chromatographic technique (Figure 5). The sensitive study revealed that the IC_50_ (11.83 μg/kg), the LOD (0.91 μg/kg), and the LOQ (2.35 μg/kg) in the real samples can fully meet the current requirements for a rapid and reliable EC determination.

#### 3.6.2. Specificity

The specificity of the immunoassay was calculated by the CR values. In Table 5, although the CR value of methyl carbamate was slightly higher, the concentration of this substance in foodstuffs was often found to be relatively low [9]. Therefore, its cross-reaction to antibodies can be ignored, indicating that the specific evaluation results of the method were acceptable.

#### 3.6.3. Accuracy and Precision

For accuracy and precision, the mean recovery rates at different levels were from 86.0% to 105.5%, with all the CVs less than 10%, indicating that the results could completely meet the corresponding requirements.

### 3.7. Detection of Real Samples

**Figure 6:**
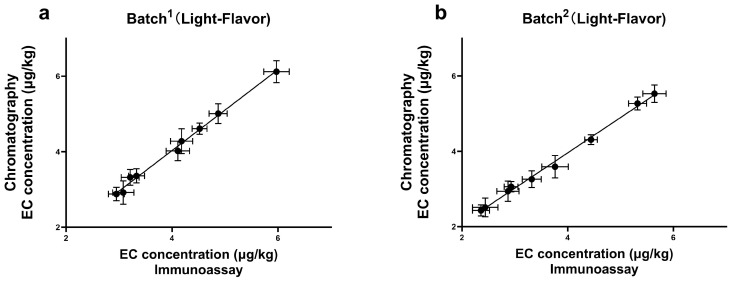

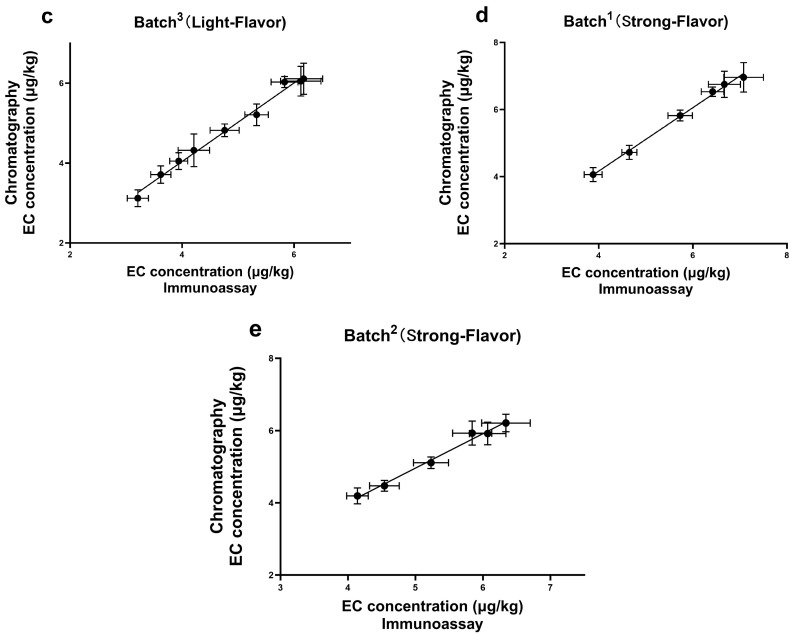
The correlation results of EC detection by ELISA and GC-MS in real samples of (**a**) batch^1^ of Light-Flavor; (**b**) batch^2^ of Light-Flavor; (**c**) batch^3^ of Light-Flavor; (**d**) batch^1^ of Strong-Flavor; (**e**) batch^2^ of Strong-Flavor. “Light-Flavor” and “Strong-Flavor” categorize different types of Baijiu, and the duration of fermentation for the initial product is quicker, resulting in a more refreshing flavor profile.

To test the feasibility of the newly established method, a total of 39 samples were collected from Baijiu industry suppliers, including Jiupei and Jiuzao ones. A parallel analysis was performed in these samples, both by the ELISA and the confirmatory method, GC-MS. According to the results (Figure 6), a good correlation was achieved (R^2^ > 0.99), and the absence of false-negative and false-positive results suggested that this method enabled quick screening and reliable quantification for Jiupei and Jiuzao samples. Furthermore, although various mitigation practices had already been implemented to control EC formation in the whole chain of Baiji, the presence of EC at a level which exceeded the LOD was still observed in all the samples. The detection range of EC was from 2.36 μg/kg to 7.08 μg/kg, with the highest concentration of 7.08 μg/kg in the Jiupei sample. Additionally, the content of EC increased during the fermentation period of Baijiu, which was consistent with the previous literature [11]. Many factors involving in the fermentation and distillation of Baijiu can contribute to EC formation, such as high temperature, pH value, and some strains of bacteria and yeast [11,12]. Our study indicates that the contamination of EC during Baijiu production still exists, and should not be neglected.

### 3.8. Comparison with Other Immunoassays

The commonly used immunoassays for EC detection in different matrices are summarized in Table 6. Because of the lack of a complex structure, the making of high-quality antibody towards EC is essential for the sensitivity and specificity of the established methods. Some novel nanoparticles were designed to improve the performance of the corresponding methods, but these strategies might not be preferable for on-site and high-throughput sample determination, and could greatly increase the cost of the analysis. Moreover, the analyzed matrices are often limited among liquid foodstuffs, such as alcoholic beverages. Still, studies focused on the fermented materials from the alcohol industry have not been developed so far. Overall, our proposed method has provided a promising strategy for fast, sensitive, and reliable quantification of EC in the complex matrices.

## 4. Conclusions

In conclusion, we have developed a fast, uncomplicated, and sensitive ELISA strategy for EC detection in by-products from the Baijiu industry. To produce the high-sensitivity and high-specificity monoclonal antibody, a hapten containing both the specific structure towards EC and the aromatic benzene ring was designed and identified. Based on the convenient sample pretreatment process, an indirect competitive ELISA system was constructed, and the crucial parameters for the analytical performance were evaluated as well. The proposed method presented satisfactory sensitivity and precision. The IC_50_, the LOD, and the LOQ could completely meet the analytical target. The newly developed method was successfully applied in real samples, and the results were confirmed by GC-MS. In general, this method has provided a practical approach to conveniently quantify EC content in the complex matrices from Baijiu liquor. EC contamination in by-products from the Baijiu industry deserves more concern and further exploration. However, the number and variety of samples collected have areas for improvement to some extent. In the future, when conditions permit, more diverse samples covering a wider geographical area can be collected for testing.

## Figures and Tables

**Table 1:** The impact of different alcohol concentrations on the analytical performance (*n* = 6).

Alcohol Concentrations (%)	Low Level			High Level		
	Recoveries (%)	RSD (%)	Inhibitory Rate (%)	Recoveries (%)	RSD (%)	Inhibitory Rate (%)
2%	94.27	6.44	30.22	107.72	3.54	68.25
4%	89.25	6.50	29.65	92.21	2.28	67.89
20%	92.68	2.24	35.44	104.21	4.16	66.68
40%	85.90	3.58	32.11	90.43	3.60	67.42

**Table 2:** The optimal dilution rate for the coating antigen and primary antibody (*n* = 6).

Dilution Rate of Primary Antibody	Dilution Rate of Coating Antigen							
	1:5000		1:20,000		1:40,000		1:100,000	
	A_max_	Inhibition Rate (%)	A_max_	Inhibition Rate (%)	A_max_	Inhibition Rate (%)	A_max_	Inhibition Rate (%)
1:5000	2.85	12	2.26	10	2.32	8	1.56	28
1:10,000	2.33	18	1.84	14	1.72	54	1.41	64
1:50,000	2.08	48	1.63	58	1.78	75	1.23	77
1:100,000	1.56	57	1.28	59	1.15	78	0.83	81

**Table 3:** The evaluation of different dilution rates of peroxidase-labeled goat anti-mouse IgG (*n* = 6).

Analytical Performance Parameters	Dilution Rate of IgG		
	1:1000	1:2000	1:5000
A_max_	1.98	2.12	1.35
IC_50_ (μg/kg)	6.21	11.20	9.12
Linear (R^2^)	0.9932	0.9711	0.9833

**Table 4:** The evaluation of different consumption rates of the sample/standard and the primary antibody (*n* = 6).

Analytical Performance Parameters	Ratios			
	20:80	40:60	50:50	70:30
A_max_	2.61	2.02	2.14	1.53
IC_50_ (μg/kg)	4.12	4.33	4.65	12.56
Linear (R^2^)	0.9812	0.9914	0.9923	0.9654

**Table 5:** The specific evaluation of the EC analogs (*n* = 6).

EC and Its Analogs	Chemical Structure	Cross-Reactivity (%)
EC	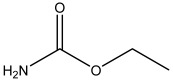	100
Methyl carbamate	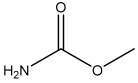	10.6
Acrylamide	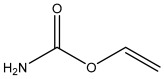	6.5
Butyl carbamate	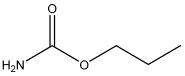	8.8

**Table 6:** Comparison of different immunoassay techniques for EC determination.

Method	Sample	Sample Pretreatment	Cut-Off Value/LOD	LOQ	Reference
Indirect ELISA	Chinese rice wine	Derivatization (ethyl carbamate with 9-xanthydrol)	166 μg/L	/ ^a^	[17]
Non-competitive ELISA	Wine	Different phages that recognize XEC were constructed	5.4 ng/mL	/	[19]
Dot-based fluorescence immunoassay	Chinese baijiu; Wine; Chinese rice wine; Brandy	Alkaline phosphatase-triggered Cu^+^ quenching of CdSe quantum dots (QDs)	24.3 ng/mL	/	[24]
Silicon-based fluorescence ELISA	Red wine	A simple pre-analysis derivatization	2.6 μg/L		[18]
Indirect competitive ELISA	By-products from the Baijiu industry		IC_50_: 11.83 μg/kg; IC_20_: 2.35 μg/kg		The proposed method

^a^: not mentioned in the literature.

## Data Availability

The original contributions presented in the study are included in the article, further inquiries can be directed to the corresponding author.

## Appendix A

The following description utilizes HPLC, LC-MS, and ^1^H NMR techniques for the specific characterization of the synthetic hapten towards EC in our study. Refer to Figure A1, Figure A2 and Figure A3 for visual representations.

HPLC: Modified hapten 2-((ethoxycarbonyl)amino)-2-phenylacetic acid) was tested for purity by UPLC as follows: ACQUITY Premier system; BEH C_18_ column (d = 1.7 μm × 2.1 mm × 5 mm), mobile phase (A is 0.1% formic acid in water, B is 0.1% formic acid in acetonitrile, A:B = 1:1), the compounds were injected into the sample in 1 μL after the concentration of 1 μg/mL was configured with mobile phase A:B = 1:1. The compounds were injected into the sample at 1 μL with the mobile phase A:B = 1:1 to a concentration of 1 μg/mL, and the flow rate was 0.4 mL/min. The first peak represented methanol, while the second peak corresponded to the purity of the product after methanol recrystallization, as shown in Figure A1, with a purity exceeding 99%.

LC-MS: For LC-MS trees, conditions are essentially similar to those of HPLC. At the retention time of 1.4 min, where the compound peaked, two numerical values for the mass-to-charge ratio (*m*/*z*) were observed. One value, 210, corresponded to the molecular ion peak, aligning with the respective molecular weight of the compound. The other value, 232, was speculated to be the dehydrogenation with sodium adduct peak.

^1^H NMR: ^1^H NMR (300 MHz, DMSO-*d_6_*) δ12.63 (br, 1H, OH), 7.40–7.25 (m, 5H, ph-H), 6.62, 6.46 (2br, 2H, NH_2_), 4.36 (dd, *J* = 6.0 Hz, *J* = 5.8 Hz, 1H), 4.08 (t, *J* = 6.0 Hz, 1H), 3.84 (d, *J* = 6.0 Hz, 1H).
